# FKBPL: a marker of good prognosis in breast cancer

**DOI:** 10.18632/oncotarget.3528

**Published:** 2015-04-03

**Authors:** Laura Nelson, Hayley D. McKeen, Andrea Marshall, Laoighse Mulrane, Jane Starczynski, Sarah J. Storr, Fiona Lanigan, Christopher Byrne, Ken Arthur, Shauna Hegarty, Ahlam Abdunnabi Ali, Fiona Furlong, Helen O. McCarthy, Ian O. Ellis, Andrew R. Green, Emad Rakha, Leonie Young, Ian Kunkler, Jeremy Thomas, Wilma Jack, David Cameron, Karin Jirström, Anita Yakkundi, Lana McClements, Stewart G. Martin, William M. Gallagher, Janet Dunn, John Bartlett, Darran O’Connor, Tracy Robson

**Affiliations:** ^1^ School of Pharmacy, Queen's University Belfast, Belfast, United Kingdom; ^2^ Warwick Clinical Trials Unit, University of Warwick, Coventry, United Kingdom; ^3^ Conway Institute, University College Dublin, Dublin, Ireland; ^4^ Ontario Institute for Cancer Research, Toronto, Canada; ^5^ Division of Cancer and Stem Cells, School of Medicine, University of Nottingham, Nottingham, United Kingdom; ^6^ Royal College of Surgeons Ireland, Dublin, Ireland; ^7^ Northern Ireland Molecular Pathology Laboratory, CCRCB, Queens University Belfast, Belfast, United Kingdom; ^8^ Department of Pathology, Royal Group of Hospitals, Grosvenor Road, Belfast, United Kingdom; ^9^ Edinburgh Cancer Research Centre, The University of Edinburgh, Edinburgh, United Kingdom; ^10^ Edinburgh Breast Unit, The University of Edinburgh, Edinburgh, United Kingdom; ^11^ Department of Clinical Sciences, Lund University, Sweden

**Keywords:** FKBPL, breast cancer, biomarker, personalized medicine

## Abstract

FK506-binding protein-like (FKBPL) has established roles as an anti-tumor protein, with a therapeutic peptide based on this protein, ALM201, shortly entering phase I/II clinical trials. Here, we evaluated FKBPL's prognostic ability in primary breast cancer tissue, represented on tissue microarrays (TMA) from 3277 women recruited into five independent retrospective studies, using immunohistochemistry (IHC). In a meta-analysis, FKBPL levels were a significant predictor of BCSS; low FKBPL levels indicated poorer breast cancer specific survival (BCSS) (hazard ratio (HR) = 1.30, 95% confidence interval (CI) 1.14–1.49, *p* < 0.001). The prognostic impact of FKBPL remained significant after adjusting for other known prognostic factors (HR = 1.25, 95% CI 1.07–1.45, *p* = 0.004). For the sub-groups of 2365 estrogen receptor (ER) positive patients and 1649 tamoxifen treated patients, FKBPL was significantly associated with BCSS (HR = 1.34, 95% CI 1.13–1.58, *p* < 0.001, and HR = 1.25, 95% CI 1.04–1.49, *p* = 0.02, respectively). A univariate analysis revealed that FKBPL was also a significant predictor of relapse free interval (RFI) within the ER positive patient group, but it was only borderline significant within the smaller tamoxifen treated patient group (HR = 1.32 95% CI 1.05–1.65, *p* = 0.02 and HR = 1.23 95% CI 0.99–1.54, *p* = 0.06, respectively). The data suggests a role for FKBPL as a prognostic factor for BCSS, with the potential to be routinely evaluated within the clinic.

## INTRODUCTION

Considerable research has been dedicated to identifying novel prognostic markers for breast cancer; however, the current panel of biomarkers available is still limited. Prognostic markers are routinely considered during disease management, with patients placed into risk groups depending upon; tumour size, grade and stage, as well as lymph-node and hormone-receptor status (reviewed in [1]). However, the ability of these markers to predict disease progression and tumor recurrence is often limited. Following the discovery of the role of estrogen in driving breast cancer development, drugs targeting estrogen receptor (ER) signalling [2, 3], i.e. endocrine therapies, have improved the survival of women with estrogen-driven breast cancer [4, 5]. Identifying patients who could benefit from endocrine therapy has become increasingly important, considering that very few women with ER-positive, node-negative tumors derive a long term benefit from endocrine therapy. The 2011 overview from the Early Breast Cancer Trialists Collaborative Group (EBCTCG) reported that with a median follow-up of 13 years [6], 5 years of tamoxifen compared to no endocrine therapy resulted in only a 9% absolute reduction in breast cancer-related death at 15 years (24% versus 33%), and the risk of breast cancer mortality was reduced by 30% (relative risk (RR) for death 0.70, 95% CI, 0.64–0.75). Some women clearly present with *de novo* resistance to tamoxifen treatment, whilst others acquire resistance [7]. Furthermore, 20–25% of patients receiving endocrine therapy discontinue treatment because of the side effects. Strategies to predict whether early stage breast cancer patients are likely to benefit from the addition of more appropriate targeted therapies or chemotherapy earlier, are needed in order to prevent further development of disease and to increase the survival rates; which patients absolutely require chemotherapy is therefore a major clinical question. The identification of additional prognostic markers could also help to identify patients who are unlikely to experience tumor recurrence and would therefore yield no therapeutic benefit from highly toxic systemic chemotherapies. Therefore, there is a need for additional prognostic markers to be developed in order to identify those patients who are likely to progress or relapse [8].

Since the mid-1970s, pathological analysis of breast tumours, usually via IHC staining, has been used to determine the ER and progesterone receptor (PR) status of the tumor, with both markers having the ability to indicate the likelihood of recurrence and response to endocrine therapies [9]. More recently, molecular assays such as Oncotype Dx (measuring 21 genes) and MammaPrint (measuring 70 genes) [10, 11], which assess risk of distant recurrence, are showing promise; however, the principle disadvantage of Oncotype DX® is the existence of a large “intermediate” group of patients for whom the correct treatment choice remains unclear. This further demonstrates the need for additional biomarkers, in order to distinguish these patients. The cheaper 5 marker IHC-based test, MammoStrat, which calculates a risk index score [12] is also showing promise, but the addition of single IHC biomarkers to standard ER/PR/Her2/Ki67 evaluation to improve sensitivity would still be advantageous, especially in terms of identifying patients who are at risk of relapse or disease progression. Recently, the PAM50 gene signature added significant prognostic information beyond the Oncotype DX^®^ Recurrence Score^®^ in estimating the likelihood of distant recurrence in hormone receptor positive, post-menopausal breast cancer patients and may help to identify women who are at high risk of late recurrence and who may benefit from either more intensive treatment (i.e. chemotherapy) or extended endocrine treatment [13–15].

FK506 binding protein like - FKBPL, is a divergent member of the immunophilin protein family. This family of proteins have wide-ranging intra- and extra- cellular roles in a host of diseases through their chaperoning function and peptidylprolyl isomerase (PPIase) activity [16]. FKBPL is clearly divergent, with no PPIase activity, whilst retaining its tetratricopeptide repeat (TPR) domain, important for the interaction with the molecular chaperone Hsp90 [17], and is emerging as a key negative regulator of tumor growth, angiogenesis and metastasis [18, 19]. We have shown previously that FKBPL is a naturally secreted anti-angiogenic protein that inhibits blood vessel development by targeting the cell surface receptor, CD44, on actively migrating endothelial cells, thereby inhibiting migration and vessel formation [19]. A ‘first-in-class’ FKBPL-based anti-angiogenic therapeutic peptide, ALM201, will shortly enter a multi-centre cancer clinical trial (EudraCT 2014–001175-31) [20]. Furthermore, we have demonstrated that the ability of FKBPL to bind CD44 makes it useful for targeting cancer stem cells (CSCs), which express high levels of CD44 [21]. FKBPL has also been implicated in steroid hormone receptor signalling due to its intracellular role in association with the molecular chaperone Hsp90 [22, 23]. We have also demonstrated the ability of RBCK1 to regulate intracellular FKBPL levels, through interaction with Hsp90 [24]. In breast cancer cells, over-expression of FKBPL resulted in reduced cell proliferation, due to stabilization of newly synthesised cyclin-dependent kinase inhibitor, p21 [25]. FKBPL levels have also been shown to correlate with sensitivity or resistance to tamoxifen therapy *in vitro*; with cells over-expressing FKBPL being more sensitive to treatment, compared with the resistance shown when FKBPL levels were reduced with FKBPL-targeted siRNA [23]. This resistance is thought to be the result of reduced p21 stabilization, which in turn leads to hyperphosphorylation of ERα at the Ser^118^ residue, altering the conformation of ERα so that tamoxifen can no longer bind [26]. Furthermore, in three of five publically available microarray datasets totalling 484 patients, high FKBPL mRNA levels were shown to correlate with improved overall survival and increased distant metastasis-free survival [23]. In addition, patients treated with tamoxifen also show a trend towards significance between high FKBPL levels and improved survival [23]. Therefore, since FKBPL demonstrates clear inhibitory roles in tumor growth and progression through a variety of independent mechanisms, its potential as a prognostic biomarker was further explored here in 5 independent TMA cohorts using IHC. An individual patient data meta-analysis was then undertaken to determine whether FKBPL could be used prognostically or had value in identifying patients most likely to benefit from tamoxifen therapy or who would be likely to benefit from adjuvant chemotherapy, particularly patients with early stage, ER+, LN-, Her2- breast cancer.

## RESULTS

### Validation to demonstrate IHC assay reproducibility, portability and application to TMAs

**Figure 1:**
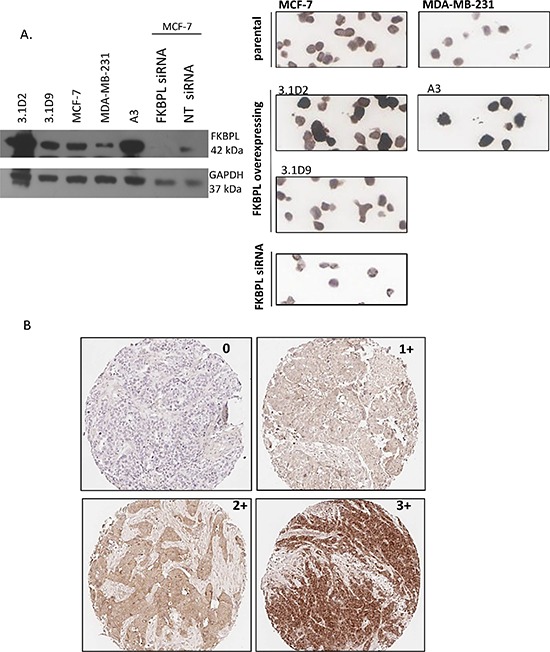
Optimisation of FKBPL antibody for IHC **(A)** Optimisation of the FKBPL anti-body for IHC staining. Specificity was verified via Western blotting, and optimized for IHC using cell pellet arrays of parental (MCF-7 and MDA-B-231) and FKBPL overexpressing (3.1D2, 3.1D9; derived from MCF-7 and A3; derived from MDA-231) cell lines or following siRNA-mediated knockdown of FKBPL in MCF-7 cells. **(B)** Various expression levels of FKBPL protein in invasive breast carcinoma. Images (x10) represent tumour sections with absent (0), low (1+), moderate (2+) and high (3+) immunohistochemical staining intensity for FKBPL.

The anti-FKBPL polyclonal antibody (Proteintech, UK) was verified for specificity via Western blotting, and optimized for IHC using cell pellet arrays of parental (MCF-7 and MDA-MB-231) and FKBPL overexpressing (3.1D2, 3.1D9; derived from MCF-7 and A3; derived from MDA-MB-231 [23]) cell lines or in MCF-7 cells where FKBPL was knocked down using an FKBPL-targeted siRNA [21] (Figure 1A). FKBPL staining intensity was clearly stronger in 3.1D2 and A3 compared to parental MCF-7 and MDA-MB-231 cells, respectively; mirroring western blot analysis. Likewise staining intensity was reduced following FKBPL-mediated siRNA knockdown compared to MCF-7 parent cells.

Full-face breast cancer (*n* = 140) and representative TMA sections obtained from Nottingham were stained and scored to ensure that TMAs were fit for purpose. For scoring FKBPL, sections/cores were assigned overall FKBPL staining intensity scores; 0, 1+, 2+ or 3+ providing over 20% of the core consisted of tumor tissue (Figure 1B). An intraclass correlation coefficient (ICCC) of 0.61 (95% confidence interval (CI) 0.49–0.70) between the full face sections and matched TMA sections for Run 1 was obtained and 0.65 (95% CI 0.55–0.74) for Run 2 (Supplementary Figure 1). These ICCCs demonstrate an acceptable measure of agreement between full face sections and the matched cores, confirming that FKBPL staining in TMA cores is representative of staining in full-face tissue sections and, therefore, that TMAs are suitable for use in the analysis of FKBPL across breast cancer TMAs [21–23].

To assess inter-lab variability, IHC staining of TMAs (*n* = 200) was carried out at three different locations, Belfast, Dublin and Toronto on three independent platforms, on consecutively cut sections and scored blindly by four independent scorers. Two staining runs were performed and independently scored by 4 individuals. The ICCC agreement between individual scorers for each staining run was 0.57 (95% CI 0.50–0.64) for run 1 and 0.62 (95% CI 0.52–0.67) for run 2, demonstrating an acceptable level of agreement between sites. The ICCC for 4 individual scorers across the two staining runs ranged from 0.61 (95% CI 0.42–0.73) to 0.90 (0.86–0.92), displaying good reproducibility. The data demonstrates reasonable assay portability as the analyses were carried out on consecutively cut sections and stained on three independent platforms. After the third and final training session for the staining and scoring methodology for FKBPL analysis, the ICCC between scorers reached 0.8 (95% CI 0.77–0.82), suggesting good correlation between scorers [36].

The reliability of manual IHC evaluation of FKBPL staining with image analysis software to quantitatively score FKBPL cytoplasmic staining was assessed within 263 tumour cores from cohort I. The median automated scores tended to increase as the manual scoring groups increased, although there appeared to be considerable overlap between the automated and manual scores with reasonable ICCCs for both scorers (Supplementary Figure 2), suggesting reasonable agreement between automated and manual scoring.

**Figure 2:**
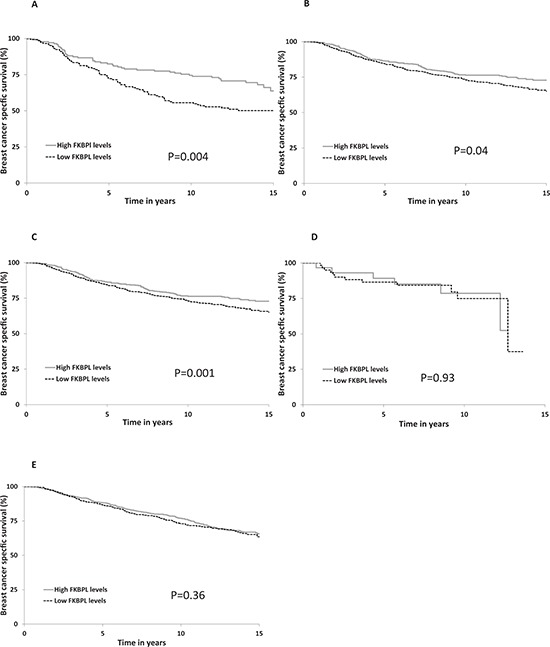
FKBPL expression Kaplan-Meier estimates of breast cancer specific survival for FKBPL in (A) cohort I (*n* = 290) (B) cohort II (*n* = 1214) (C) cohort III (*n* = 492) (D) cohort IV (*n* = 104) and (E) cohort V (*n* = 1169) FKBPL histoscore was categorised into high and low groups using a cut-point of 190.

### Evaluation of FKBPL expression and breast cancer specific survival

An individual patient data meta-analysis was carried out using five TMA cohorts in order to fully determine the ability of FKBPL to predict outcome across the whole patient cohort (*n* = 3277). The patient clinico-pathological variables are shown for all five cohorts (Table 1), with the distribution of variables remaining similar throughout. The median FKBPL histoscore value over the whole 5 cohorts was 190 (interquartile range 150–200). The median FKBPL value for cohort IV of 156 was far lower than that of the other cohorts; with two thirds of patients with low FKBPL levels in cohort IV (Table 1).

**Table 1:** Associations between clinico-pathological criteria in breast cancer samples across the five cohorts included in the meta-analysis

	Cohort I	Cohort II	Cohort III	Cohort IV	Cohort V
*N*	290	1214	492	112	1169
**Characteristics**					
**Treated with tamoxifen**	140 (48%)	445 (37%)	157 (32%)	67 (60%)	840 (72%)
**FKBPL histoscore**					
**Median (IQR)**	187 (122–200)	180 (165–200)	200 (150–200)	156 (117–200)	200 (150–200)
**Low**	146 (50%)	728 (60%)	216 (44%)	76 (68%)	400 (34%)
**High**	144 (50%)	486 (40%)	276 (56%)	36 (32%)	769 (66%)
**Tumor size**					
**< 20 mm**	100 (34%)	592 (49%)	306 (62%)	30 (27%)	417 (36%)
**>= 20 mm**	189 (65%)	615 (50%)	186 (38%)	60 (53%)	712 (61%)
**Unknown**	1 (1%)	7 (1%)	0	22 (20%)	40 (3%)
**Tumor grade**					
**1**	34 (12%)	211 (17%)	123 (25%)	6 (6%)	253 (22%)
**2**	112 (39%)	412 (34%)	203 (41%)	38 (34%)	494 (42%)
**3**	140 (48%)	584 (48%)	165 (33%)	51 (46%)	414 (35%)
**Unknown**	4 (1%)	7 (1%)	1 (1%)	17 (15%)	8 (1%)
**Nodal status**					
**Negative**	81 (28%)	639 (53%)	279 (57%)	44 (39%)	801 (68.5%)
**Positive**	207 (71%)	442 (36%)	161 (33%)	41 (37%)	367 (31.4%)
**Unknown**	2 (1%)	133 (11%)	52 (10%)	27 (24%)	1 (0.1%)
**ER status**					
**Negative**	99 (34%)	287 (24%)	73 (15%)	28 (25%)	294 (25%)
**Positive**	141 (49%)	891 (73%)	419 (85%)	69 (62%)	845 (72%)
**Unknown**	50 (17%)	36 (3%)	0	15 (13%)	30 (3%)
**PR status**					
**Negative**	88 (30%)	458 (38%)	157 (32%)	39 (35%)	245 (21%)
**Positive**	150 (52%)	687 (56%)	335 (68%)	53 (47%)	898 (77%)
**Unknown**	52 (18%)	69 (6%)	0	20 (18%)	26 (2%)
**HER2 status**					
**Not-amplified**	198 (68%)	1031 (85%)	438 (89%)	50 (45%)	812 (69%)
**Amplified**	64 (22%)	162 (13%)	42 (9%)	10 (9%)	135 (12%)
**Unknown**	28 (10%)	21 (2%)	12 (2%)	52 (46%)	222 (19%)
**Triple negative**	50 (17%)	192 (16%)	39 (8%)	13 (12%)	64 (5%)
**KI67**					
**Not amplified**	111 (38%)	0	175 (36%)	0	519 (44%)
**Amplified**	151 (52%)	0	286 (58%)	0	610 (52%)
**Unknown**	28 (10%)	1214 (100%)	31 (6%)	112 (100%)	40 (4%)

Abbreviations: PR-progesterone receptor, ER-estrogen receptor, HER2- receptor tyrosine-protein kinase erbB-2, IQR – Interquartile range

**Figure 3:**
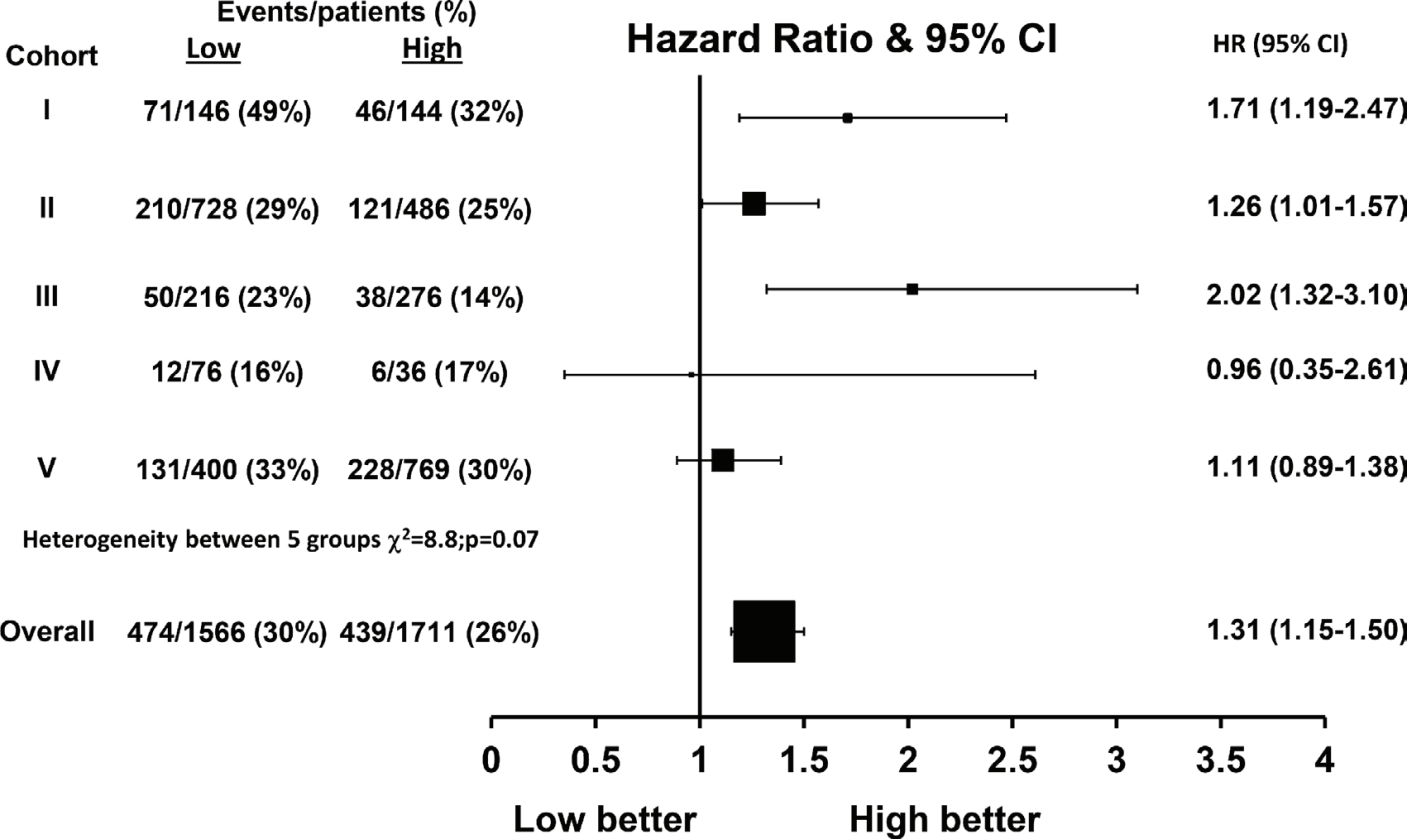
Hazard ratio plot of breast cancer specific survival against FKBPL levels by cohort using a one stage random effects meta-analysis model (*n* = 3279)

With a median follow-up of 12 years (interquartile range 10–15 years), there were a total of 913 (28%) breast cancer deaths and 1295 (40%) recurrences within the combined cohort of 3277 patients. Within the individual cohorts, breast cancer specific survival (BCSS; Figure 2) was significantly different across FKBPL expression groups within cohorts I (*p* = 0.004, Figure 2A), II (*p* = 0.04, Figure 2B) and III (*p* = 0.001, Figure 2C), but not within cohorts IV (*p* = 0.93, Figure 2D) and V (*p* = 0.36, Figure 2E) with lower FKBPL levels associated with poor BCSS (e.g. within cohort I: Hazard ratio (HR) = 1.71 (95% Confidence Interval (CI) 1.19–2.47, Figure 3). The meta-analysis of these five cohorts (*n* = 3277) was performed using a one stage random effects model, as there appeared to be some heterogeneity between the cohorts (χ^2^ = 8.8, *p* = 0.07, Figure 3). Patients with lower FKBPL levels had significantly shorter BCSS than those with higher FKBPL levels (HR = 1.31, 95% CI 1.15–1.50, *p* < 0.001, Figure 3). In a multivariate random effects analysis, the effect of FKBPL on BCSS remained significant after adjusting for other known prognostic factors, including; tumor size, grade, nodal status, ER and PR status with time dependent covariates (HR = 1.25, 95% CI 1.07–1.45, *p* = 0.004), and after the addition of Her2 status as a time dependent covariate (HR = 1.21, 95% CI 1.06–1.42, *p* = 0.02). FKBPL was also significant after adjusting for the Nottingham Prognostic Index (NPI) (HR = 1.31, 95% CI 1.14–1.50, *p* = 0.0002); which was available in cohorts I, II, III, V (>3000 patients).

### Evaluation of FKBPL expression and BCSS in endocrine therapy treated patients

**Figure 4:**
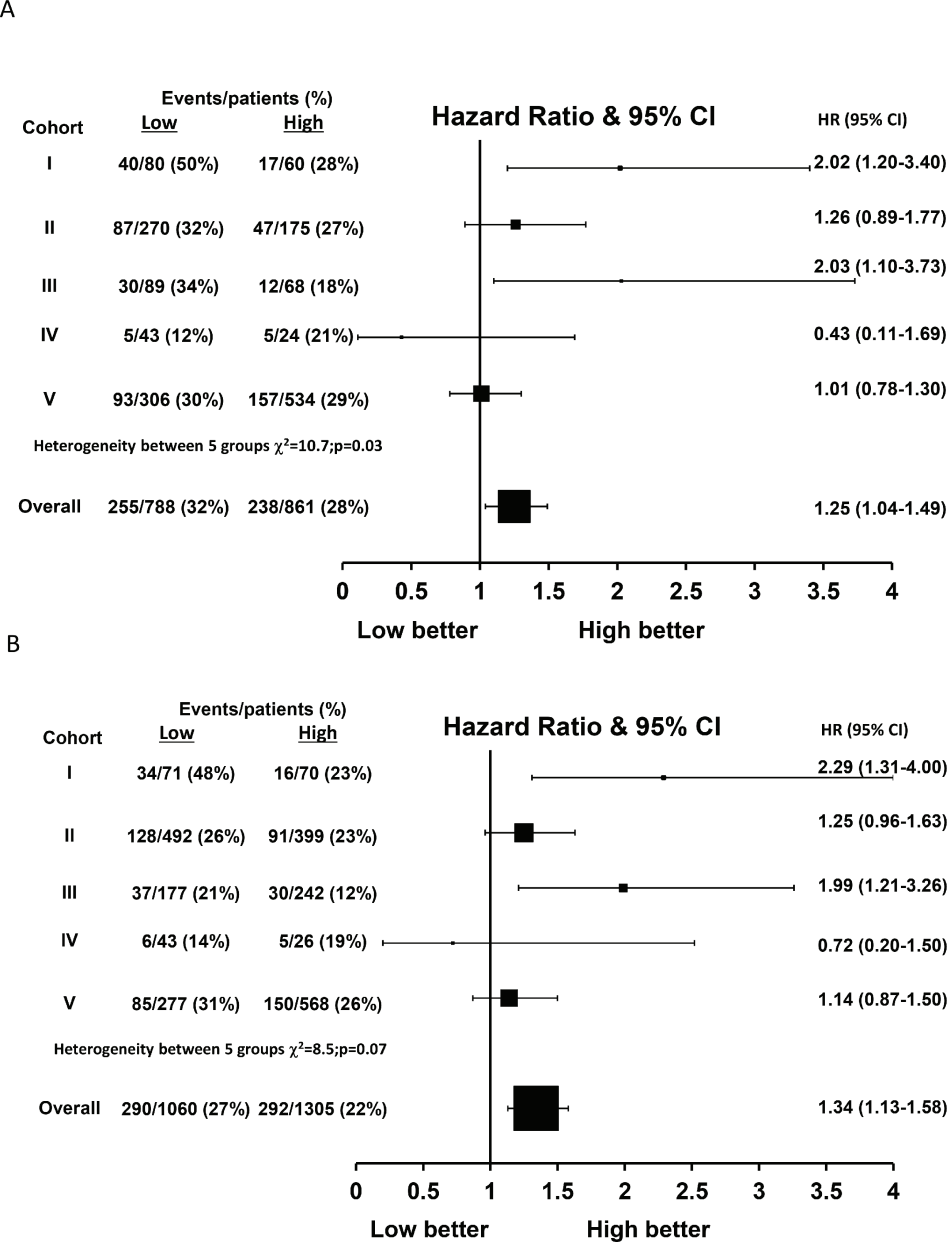
Hazard ratio plot of breast cancer specific survival against FKBPL levels by cohort using a one stage random effects meta-analysis model in (A) tamoxifen treated patients (*n* = 1649) and (B) ER-positive patients (*n* = 2365)

There was some heterogeneity between cohorts when considering the effect of FKBPL expression on BCSS within the subgroup of 1649 tamoxifen treated patients (χ^2^ = 10.7, *p* = 0.03, Figure 4A). Using a one stage random effects model, patients with lower FKBPL levels have significantly shorter BCSS than those with high expression within the tamoxifen treated population (HR = 1.25, 95% CI 1.04–1.49, *p* = 0.02, Figure 4A).

### Evaluation of FKBPL expression and BCSS in ER positive patients

Similarly, there was some evidence of heterogeneity in the effect of FKBPL on BCSS across the five cohorts for the 2365 ER positive only patients (χ^2^ = 8.5, *p* = 0.07, Figure 4B). ER positive patients with low FKBPL expression had a significantly reduced BCSS compared with patients with high FKBPL levels (HR = 1.34, 95% CI 1.13–1.58, *p* < 0.001, Figure 4B). FKBPL was a significant predictor of BCSS in the subgroup of 834 ER positive, node positive patients (HR = 1.41, 95% CI 1.12–1.77, *p* = 0.004). However, there was a borderline prognostic effect of FKBPL in the 1361 ER positive, node negative patients (HR = 1.27, 95% CI 0.98–1.65, *p* = 0.07) and a significant effect within the subgroup of 1148 ER positive, node negative, HER2 negative patients (HR = 1.35, 95% CI 1.01–1.79, *p* = 0.04).

### Evaluation of FKBPL expression and RFI in tamoxifen treated and ER positive patients

**Figure 5:**
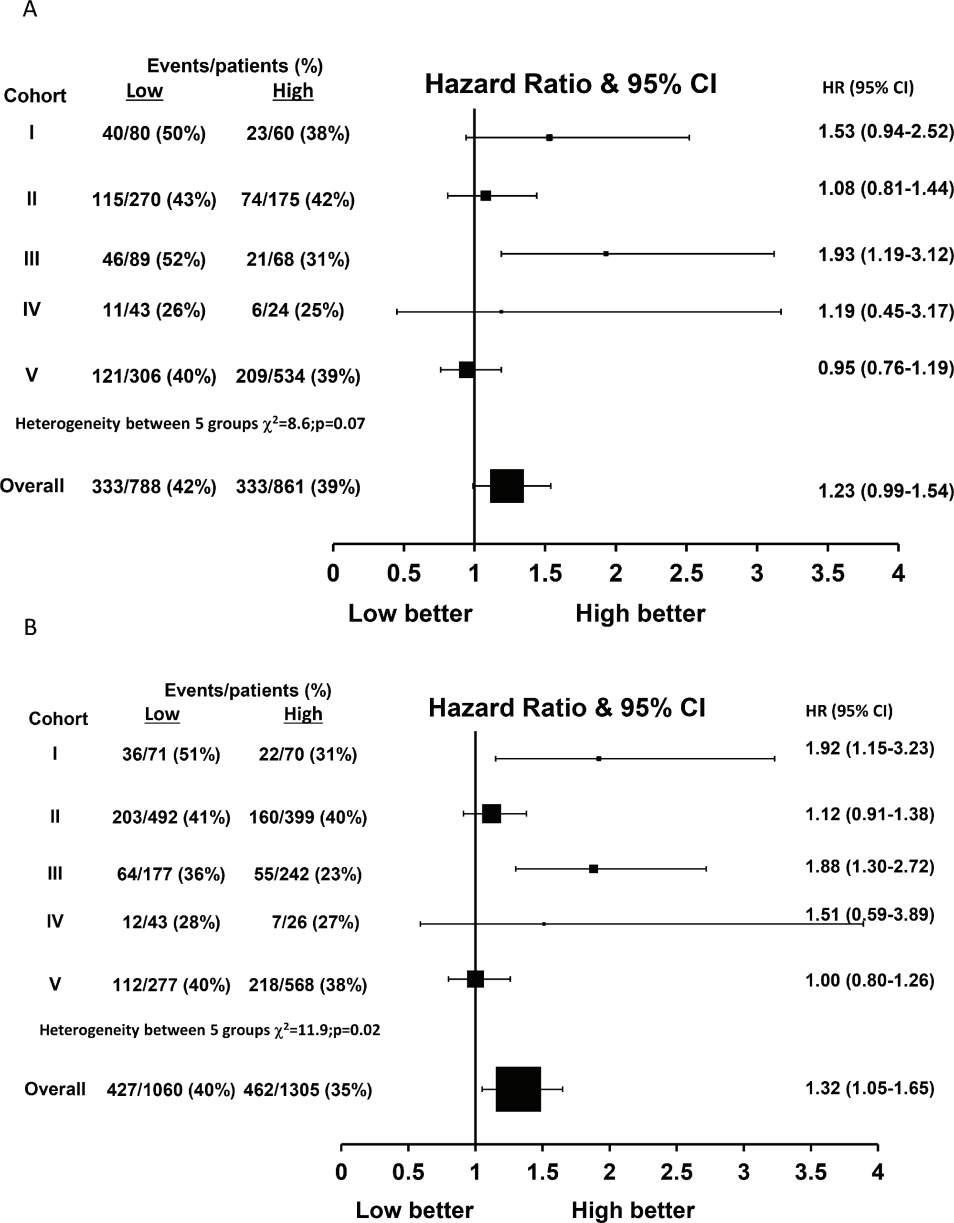
Hazard ratio plot of relapse free interval against FKBPL levels by cohort using a one stage random effects meta-analysis model in (A) tamoxifen treated patients (*n* = 1649) and (B) ER-positive patients (*n* = 2365)

Within the 1649 tamoxifen treated patients, FKBPL expression was only able to predict outcome in tamoxifen-treated patients for RFI within cohort III (*p* = 0.007) but not within cohorts I, II, IV and V (*p* = 0.09, 0.60, 0.73 and 0.65 respectively, Figure 5A). Over all five cohorts, FKBPL expression was a borderline significant predictor of RFI in a random effects model (HR = 1.23, 95% CI 0.99–1.54, *p* = 0.06, Figure 5A). However, FKBPL was significantly associated with RFI within the 2365 ER positive patients (HR = 1.32, 95% CI 1.05–1.65, *p* = 0.02, Figure 5B).

The interaction of tamoxifen treatment and FKBPL was assessed within the 290 cohort I patients that were randomised between 2 years tamoxifen and observation, but the interaction term was not significant for predicting RFI (*p* = 0.46). In addition, there was no significant interaction between the use of tamoxifen treatment and FKBPL expression for RFI in all patients (*p* = 0.38).

### Effects of TMA core loss on patient distribution

The characteristics of the patients with or without sample availability were similar and a sensitivity analysis using multiple imputations produced the same conclusions (data not shown).

## DISCUSSION

The recent advances in gene expression profiling suggest an increasingly important role for its use in disease management [41, 42]. Despite these advances, there are a number of limitations, such as high cost, which mean that biomarker analysis using IHC remains the most commonly used method for tailoring cancer therapy [43]. Currently, there is a very limited panel of commonly used biomarkers; therefore it is essential to identify novel biomarkers, to further stratify patients with respect to prognosis or response to treatment.

This paper investigates the potential of FKBPL as a prognostic marker in breast cancer patients. We have previously presented data demonstrating an in *vitro* role for FKBPL in association with HSP90, where it has been associated with cell proliferation and sensitivity to endocrine therapies [23, 25, 26]. FKBPL mRNA levels were also previously shown to significantly prognosticate patient outcome, whilst there was a trend towards significance in relation to response to tamoxifen therapy [23]. Furthermore, loss of the region of chromosome 6, on which FKBPL is located, has been shown to occur more frequently in patients presenting with cancer recurrence within 5 years of diagnosis [44].

Our previous work has already demonstrated an association between FKBPL and survival in cohort III [32]; here, we evaluated four other cohorts (I, II, IV and V) using a systematic and standardised approach. FKBPL was clearly associated with BCSS in cohorts I (*P* = 0.004) and II (*p* = 0.04) and when cohort III (*p* = 0.001) was reanalysed using a histoscore rather than staining intensity, an association was still observed. Histoscores were used so that statistical evaluation of FKBPL levels across all cohorts could be standardised and where percentage tumour stained could also be considered; thus allowing FKBPL to be categorised into low and high levels according to the median value of 190, across all cohorts. In cohorts IV and V, FKBPL did not significantly correlate with BCSS (*p* = 0.93 and *p* = 0.36 respectively). The variation between cohorts was probably due to small sample numbers available in cohort IV, and a poor distribution of intensity score in cohort V; both viable reasons for a lack of significance within these cohorts. Furthermore, we note that Cohort V had a higher proportion of patients with a positive PR status which may be affecting the high proportion of patients with high FKBPL levels. In addition, a larger proportion of patients were treated with tamoxifen (both ER positive and ER negatives) in Cohort V; it is difficult at this stage to determine whether these differences could impact on FKBPL's prognostic ability within this cohort. However, with the significant increase in power achieved through the meta-analysis of all 3277 patients from the five cohorts, we were able to demonstrate that BCSS significantly differs across FKBPL levels, with low FKBPL expression having shorter BCSS (HR = 1.31, 95% CI 1.15–1.50, *p* < 0.001).

Interestingly, other members of the FK506-binding protein family (FKBPs) have also been implicated in cancer progression and have shown potential as cancer biomarkers. FKBP12 has been shown to stabilize the inactive form of tumor growth factor β (TGF-β) receptor 1, inhibiting cell growth and differentiation, as well as apoptosis [45]. However unlike FKBPL, whose over expression has been shown to significantly improve overall survival, FKBP12 over expression is associated with a less favorable outcome. Whilst screening for angiogenesis-related proteins in malignant high-grade astrocytomas, FKBP12 was shown to be one of 17 genes upregulated [46].

FKBP51 is another FKBP which has been shown to play a role in steroid hormone signalling, where it displays both inhibitory [47] and stimulatory [48] roles. Furthermore, FKBP51 has also been associated with chemo- and radio-resistance in various cancers, including breast cancer. This is thought to be a result of FKBP51′s ability to dephosphorylate Akt, a protein kinase which promotes cell survival and inhibits apoptosis [49]. Additionally, other published *in vitro* analyses have demonstrated that decreased FKBP51 expression resulted in chemo-resistance in a variety of cancer cell lines, with these findings expected to translate into a clinical setting [50]. These findings are in line with our findings for FKBPL.

Based on previously published data [23], a role for FKBPL in prognosticating patient survival following endocrine therapy was anticipated. FKBPL has previously been shown to modulate cell sensitivity to tamoxifen treatment, with clonogenic survival of MCF-7 cells over expressing FKBPL being significantly less than the MCF-7 control. Following the meta-analysis, a significant association between high FKBPL expression and BCSS in patients treated with endocrine therapy was observed but not for RFI at this stage, as there were still too few events. The predictive potential of FKBPL was also examined within cohort I, where patients had either received tamoxifen treatment, or not. We were unable to see any significant correlation between FKBPL expression and response to tamoxifen therapy. It is therefore important to further explore the predictive ability of FKBPL within a larger patient population.

A major requirement of new biomarkers is their ability to function independently. Therefore multivariate analysis was carried out to determine whether the prognostic ability of FKBPL remained significant after adjusting for current markers. Our data demonstrates after adjusting for tumor size, grade, nodal status, ER and PR status and also HER2 status, FKBPL remains significant. Significance was also maintained after adjusting for NPI in four or the five cohorts, again suggesting that FKBPL adds additional prognostic information above current tools. Furthermore, the prognostic effect of FKBPL in the 1361 ER positive, node negative patients was borderline significant (*p* = 0.07) and significant within the subgroup of 1148 ER positive, node negative, HER2 negative patients (*p* = 0.04). Currently, ER-positive, node-negative patients would be assigned to tamoxifen therapy. However, this data suggests that ER-positive, node-negative patients, who also have low FKBPL expression, may have aggressive tumours and therefore benefit from adjuvant chemotherapy alongside tamoxifen treatment. It would also be interesting to further evaluate FKBPL's association with outcome by determining the effects of differential FKBPL expression on response to different treatment regimes, for example endocrine therapy alone or in combination with chemotherapy. Unfortunately, the number of patients receiving a combination of chemo- and endocrine therapy within this analysis was too small (*n* = 164).

We have demonstrated a role for FKBPL in prognosticating BCSS within a large meta-analysis; however its ability to influence clinical management requires further investigation. Nevertheless, the data generated through this collectively large patient cohort support the emerging *in vitro* and *in vivo* data highlighting FKBPL's anti-tumor activity [18, 19, 21, 24]. FKBPL has known anti-angiogenic [18, 19] and anti-cancer stem cell activity [21] and its over expression can slow the growth of breast tumors through stabilisation of p21 [25], so it's prognostic ability is not surprising. In summary, we have demonstrated that our FKBPL IHC-biomarker is fit for purpose, portable and reproducible across laboratories, and could be scored using our automated algorithm that may allow for more precise cut-offs in decision making across these sites, in the future. Furthermore, these data provide support for the use of the FKBPL biomarker as a prognostic aid to patient management in early-stage ER+, LN-, Her2- breast cancer, which could be easily and cheaply incorporated alongside these standard biomarkers. Currently, this subgroup of patients would usually receive endocrine therapy alone. However, our data suggest that if FKBPL expression is low, their survival is worse than those with high FKBPL expression and therefore these patients may benefit from the addition of adjuvant chemotherapy.

## MATERIALS AND METHODS

### Patient characteristics

Individual patient data from five cohorts were obtained. These cohorts were selected as they were derived from breast cancer patients which had similar clinico-pathological features and patients had received similar treatment regimen. Data requested from all cohorts included survival and relapse information, endocrine treatment, and the characteristics of tumor size and grade, lymph node status, ER status, PR status, Her2 status and Ki67 where available.

*Cohort I- Randomised control trial*: The Swedish Randomised Study of 2 Years of Adjuvant Tamoxifen vs No Treatment recruited 564 patients between 1984 and 1991. These were either premenopausal or under 50 years with stage 2 invasive breast cancer, treated by radical mastectomy or breast-conserving surgery including axillary node clearance [27]. Those suitable for breast-conservation were treated with radiotherapy (50Gy), whilst patients with axillary node metastases received additional regional radiotherapy. Patients were randomly assigned between groups, with 288 patients in the control arm who received no treatment and 276 patients receiving 2 years of adjuvant tamoxifen, 20–40 mg daily. Less than 2% (*n* = 9) of patients received adjuvant poly-chemotherapy. The aim of this clinical trial was to examine the effect of tamoxifen on recurrence free survival (RFS) and the study has been described in detail elsewhere, including as part of the Oxford meta-analysis [28, 29]. The study was approved by the ethical committees at Lund and Linköping Universities. Of the 440 patients represented on the TMAs, only 290 patients were available for analysis due to core loss during sectioning and staining.

*Cohort II- Nottingham cohort*: This is a well-characterised consecutive series of patients treated according to standard clinical protocols and has been reported upon previously [30]. The cohort consisted of 1902 early stage invasive breast cancer patients treated at Nottingham University Hospitals, between 1987 and 1998. The median age of patients was 55 years (range 18–72) with a high proportion having stage 1 disease (1203/1902). All patients underwent a mastectomy or wide local excision, followed by radiotherapy if indicated. Patients received systemic adjuvant treatment on the basis of the NPI, ER and menopausal status. Patients with an NPI value of less than 3.4 did not receive adjuvant therapy and patients with an NPI value of 3.4 were candidates for cyclophosphamide, methotrexate and 5-fluorouracil chemotherapy if they were ER negative or premenopausal and for endocrine therapy if they were ER positive. Endocrine therapy included either tamoxifen, or a combination of tamoxifen and goserelin acetate. Of the 1902 patients, 1214 patients were available for analysis due to core loss during sectioning and staining or there being insufficient tumor to score accurately.

In addition, a training TMA (*n* = 200) from Nottingham was also used to assess suitability of TMAs against matched full face breast cancer sections on two separate runs and to assess inter-lab variability of staining and scoring.

*Cohort III- Swedish Malmö Cohort*: The Swedish Malmö cohort consisted of 512 consecutive breast cancer cases diagnosed at the Department of Pathology, Malmö University Hospital, Malmö, Sweden, between 1988 and 1992 and has been described previously [31]. The median age was 65 years (range 27 – 96). Complete treatment data were available for 379 (76%) patients, 160 of whom had received adjuvant tamoxifen. Twenty three patients received adjuvant chemotherapy. Two hundred patients received no adjuvant systemic treatment. All invasive TNM stages were represented within the cohort. Five hundred of 512 patients were represented on TMAs, with 492 cores being scoreable. Survival analysis for this cohort has been previously published and reported using FKBPL staining intensity rather than histoscore [32]; alternative cut-offs were used here.

*Cohort IV- Waterford retrospective cohort*: Tissue was obtained as part of a retrospective study from the Waterford Regional Hospital between 1998 and 2004. The median age was 56 years (range 26 to 84). The cohort consisted of 292 patients treated with radical mastectomy or breast-conserving surgery including axillary node sampling and clearance. Those suitable for breast-conservation were treated with radiotherapy. Excluded from the analysis were patients who did not have breast surgery, those who had neoadjuvant therapy, or those whose tissue specimens were irretrievable. Follow-up data, average 8.4 years, was collected on the patients to determine disease free survival (DFS) and overall survival. One hundred and twelve patients were available for analysis where FKBPL was measured and 87 of them were ER positive and received endocrine treatment.

*Cohort V- Breast Conservation Surgery (BCS) cohort*: Tissue obtained from the Edinburgh BCS comprised of a consecutive cohort of 1812 patients treated by breast conservation surgery, axillary node sampling or clearance, and whole breast radiotherapy between 1981 and 1998. Patients were those considered suitable for breast-conserving therapy and were T1 or T2 (<30 mm), N0 or N1 and M0 for conventional tumour node metastasis staging. Post-operative breast radiotherapy was given over 4–5 weeks at a dose of 45 Gy in 20–25 fractions. Patients received adjuvant systemic therapy as follows: tamoxifen, other endocrine therapy, chemotherapy alone, chemotherapy plus endocrine therapy, no adjuvant systemic therapy. A total of 1169 patients were available for analysis due to variable core loss between centers, which resulted from extensive use of TMAs in previous studies.

### Tissue microarray construction

All TMAs were constructed at each of the various centres; cohort I-Lund University [33], cohort II- Nottingham University [30], cohort III- Malmö University Hospital [31], cohort IV- Waterford Regional Hospital and cohort V- Edinburgh Breast Unit [34]. In brief, TMAs were constructed using standard formalin-fixed, paraffin-embedded tissue sections of primary breast carcinoma. Single (cohort II and cohort III), duplicate (cohort I), triplicate (cohort V) and quadruplicate (cohort IV) cylindrical cores with a diameter of 0.6 mm were taken from areas showing tumor on slides stained for haematoxylin and eosin. The number of cores on the recipient blocks varied between cohorts.

### Immunohistochemistry staining of tissue

Tissue staining was carried out in various locations, as follows. For optimisation of staining and scoring, this was carried out at the three main centres, Belfast, Toronto and Dublin as described below. Cohort I was stained at the Northern Ireland Molecular Pathology Laboratory of Queen's University Belfast, Centre for Cancer Research and Cell Biology, using the Ventana Discovery XT Immunostainer (VentanaMedical Systems Inc, Arizona, USA). Standard IHC techniques were used to stain the TMA with the FKBPL antibody (1:800), with an incubation period of 1 h. Cohort II was stained manually, following antibody optimisations, at the University of Nottingham. Following initial processing, FKBPL antibody was added at a dilution of 1:100 for one hour and the Novolink Polymer Detection Kit (Leica) used. Positive and negative controls were included. Cohort III was stained as previously described [20]. Cohort IV was stained manually, as previously described [35], at the Royal College of Surgeons Ireland, Dublin, using an antibody dilution of 1:100 and incubation period of 1 h. Finally, cohort V was stained at the Ontario Institute of Cancer Research, Toronto, with the FKBPL antibody (1:600) using the Benchmark XT staining system (Ventana Medical Systems Inc, Arizona, USA), with an incubation period of 1 h.

For all cohorts, staining was visualised with 3, 3-diaminobenzidine (DAB) and lightly counterstained using hematoxylin. Following staining, TMAs were securely stored shielded from light at room temperature.

### Evaluation of immunohistochemical staining

In order to confirm that the staining and scoring methodology for FKBPL analysis was robust, training TMAs were constructed using a sample of tumor cores (*n* = 200) provided by Nottingham University. IHC staining of these TMAs was carried out at three different locations, Belfast, Dublin and Toronto on three independent platforms, on consecutively cut sections and scored blinded by four independent scorers. Similarly, FKBPL staining was evaluated on matched full face sections. In order to determine the reliability of manual IHC evaluation of FKBPL staining, we utilized image analysis software to quantitatively score FKBPL cytoplasmic staining within 263 tumour cores from cohort I.

TMAs were scored fully by one ‘trained’ scorer, with a second, independent scorer evaluating a minimum of 10% of the cohort. Each scorer was ‘blind’ to all pathological information, as well as the others scores. FKBPL staining was localised to the cytoplasm of tumor cells and scored according to staining intensity. Cores were assigned overall FKBPL staining intensity scores; 0, 1+, 2+ or 3+ providing over 20% of the core consisted of tumor tissue (Figure 1B). In addition, cores were also awarded the percentage of tumor stained with FKBPL and from this a histoscore was calculated, where intensity scores were multiplied by percentage of core stained, with a maximum value of 300. For generation of automated scores, high resolution digital images were captured using the ScanScope XT slide scanner (Aperio Technologies, now part of Leica Biosystems). TMA images were dearrayed and managed using Spectrum software (Aperio Technologies) and a color deconvolution algorithm (Aperio Technologies) was used to develop a quantitative scoring model of FKBPL expression. Scoring results were established prior to transfer to the independent statistics team at the University of Warwick for analysis with the clinical outcome data.

### Statistics

The FKBPL histoscores were categorised into low and high levels according to the median value of 190, across all cohorts. Agreement between scorers was assessed by ICCC, obtained from a two-way random effects model for the absolute agreement using SPSS statistical software package (IBM SPSS Statistics 19). ICCC values can range between 0–1.0, with values of 0.6 being acceptable and above 0.70 taken to confirm good agreement between scorers [36]. Similarly, ICCCs were calculated to assess agreement between the results of full face sections and manual scores on two separate occasions and a box and whisker plot constructed.

In order to compare manual FKBPL cytoplasmic scoring with automated image analysis, a box and whisker plot for the automatic scoring values was plotted against the manual score separately for each manual scorer. A random effects model accounting for the manual scoring groups was fitted to obtain an ICCC as a measure of the homogeneity of the automatic scores within manual scoring groups.

BCSS was calculated as the time from diagnosis until the date of death from breast cancer or censored at the date of death if died of other causes or the date last known to be alive. RFI was calculated as the time from diagnosis until the date of first relapse or date of breast cancer death if died without recorded relapse. Survival analyses were performed using the SAS statistical package (version 9.3). For each cohort, Kaplan-Meier survival curves were constructed for BCSS and RFI and compared across FKBPL expression groups using a log rank test.

To assess the prognostic ability of FKBPL across cohorts a hazard ratio plot was constructed and the Cochran's Q statistic was used to assess the level of heterogeneity between cohorts and the need for a random effects model [37]. A one stage random effects Cox regression model [38] was undertaken using the R statistical software (version 3.0.3) when there was some evidence of heterogeneity to assess the prognostic ability of FKBPL. The effect of FKBPL after adjusting for known prognostic factors including; tumor size, grade, nodal status, ER and PR status was also determined. Time dependent covariates were fitted when the proportional hazards assumption failed. Analyses were also performed within the tamoxifen treated patients and ER positive patients. The interaction of tamoxifen treatment and FKBPL on RFI was considered for all five cohorts and more specifically within the 290 cohort I only patients that were randomised to have two years tamoxifen versus observation.

Due to samples only being available on a proportion of patients within each cohort, a sensitivity analysis was performed to assess the stability of the results using multiple imputation using a fully conditional specification method [39] within SAS (version 9.3) statistical package with 20 imputations and the results combined using Rubin's rules [40].

## SUPPLEMENTARY FIGURES


