# Supplementary material for: FKBPL: a marker of good prognosis in breast cancer
Source: Oncotarget. 2015 Apr 3;6(14):12209–23. doi: 10.18632/oncotarget.3528 (PMC4494933; doi:10.18632/oncotarget.3528)
Supplement: Supplementary file 1 [file oncotarget-06-12209-s001.pdf]

SUPPLEMENTARY FIGURES

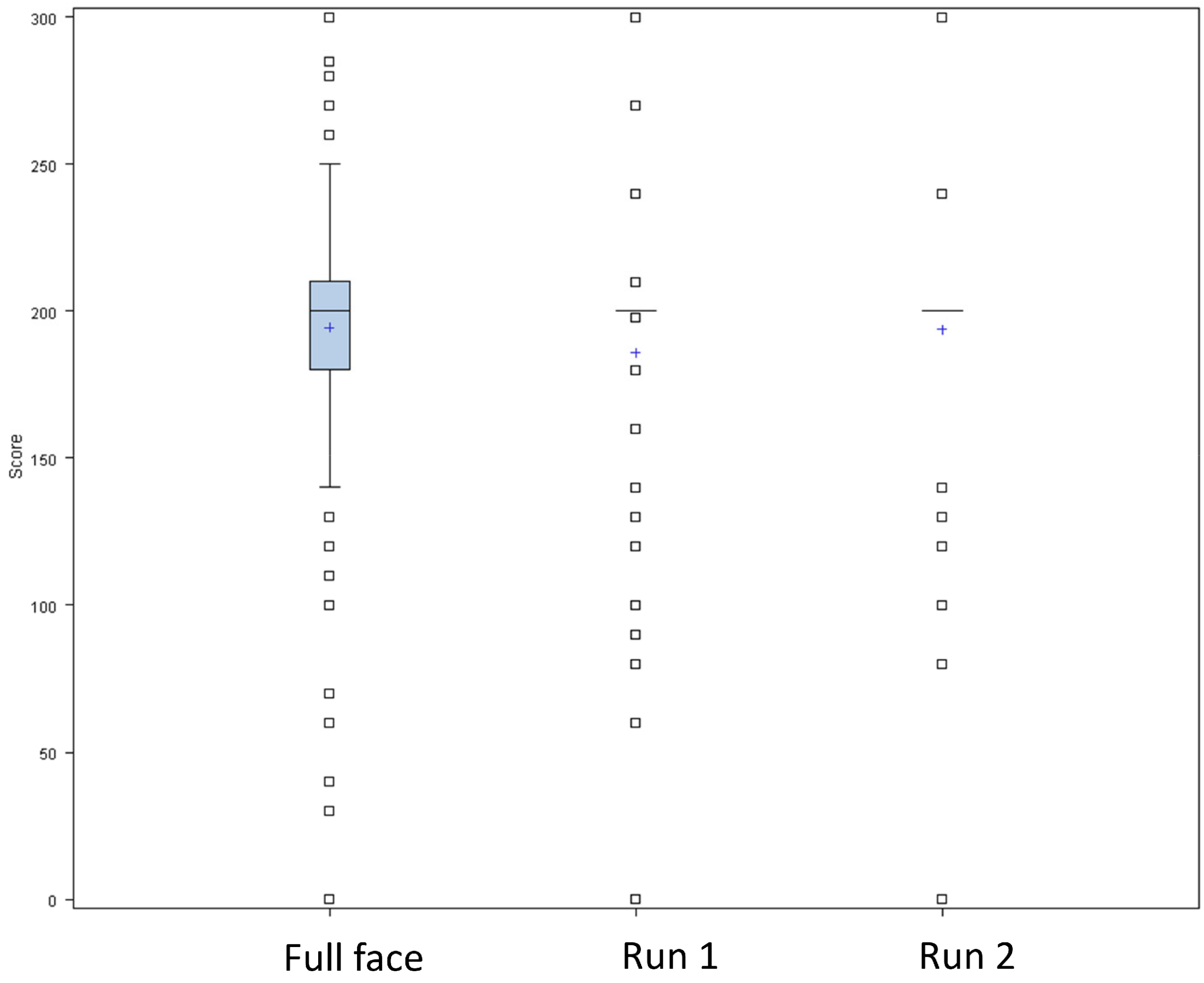

Supplementary Figure 1: Box and whisker plot of the FKBPL scores from 140 full face sections provided by Nottingham compared with scores obtained from TMAs on two separate runs.

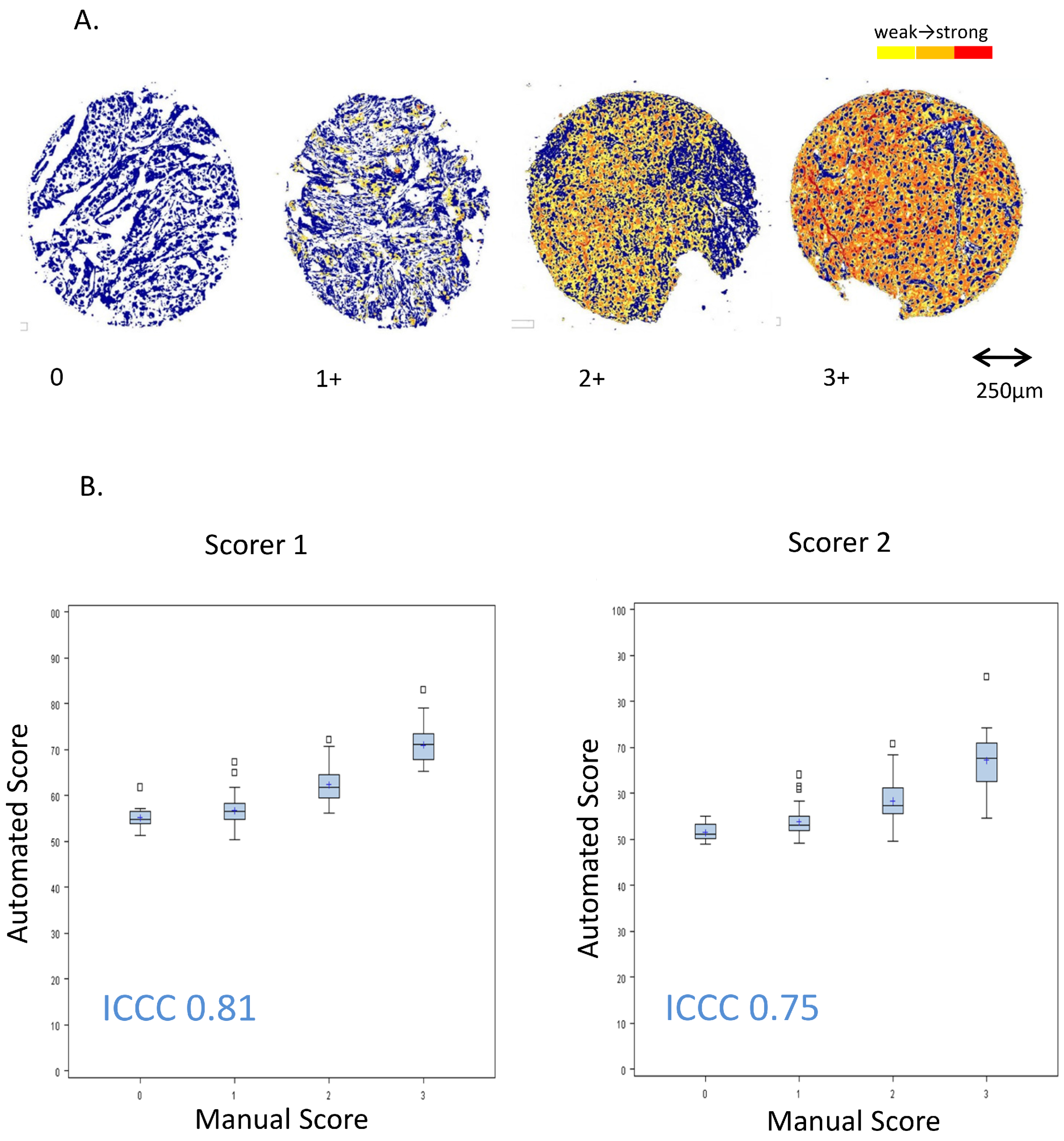

Supplementary Figure 2: Comparison of automated and manual scoring methods of FKBPL staining intensity within 263 tumour cores (cohort I). (A) Expression levels of FKBPL protein as detected by the automated system and **(B)** a box and whisker plot comparing the automated and manual scores for two scorers separately.
